# Prevalence of* Schistosoma mansoni* Infection in Four Health Areas of Kisantu Health Zone, Democratic Republic of the Congo

**DOI:** 10.1155/2016/6596095

**Published:** 2016-08-07

**Authors:** R. Khonde Kumbu, K. Mbanzulu Makola, Lu Bin

**Affiliations:** ^1^Key Laboratory of Environment and Health, Ministry of Education and Ministry of Environmental Protection and State Key Laboratory of Environmental Health (Incubating), School of Public Health, Tongji Medical College, Huazhong University of Science and Technology, Wuhan, Hubei 430030, China; ^2^Department of Pediatrics, Faculty of Medicine, University of Kinshasa, P.O. Box 747, Kinshasa, Democratic Republic of the Congo; ^3^Department of Pediatrics, Faculty of Medicine, Catholic University of Bukavu, P.O. Box 285, Bukavu, South Kivu, Democratic Republic of the Congo; ^4^Department of Tropical Medicine, Infectious and Parasitic Diseases, Faculty of Medicine, University of Kinshasa, P.O. Box 747, Kinshasa, Democratic Republic of the Congo

## Abstract

*Background*. Schistosomiasis is a public health problem in Democratic Republic of the Congo but estimates of its prevalence vary widely. The aim of this study was to determine prevalence of* Schistosoma mansoni* infection and associated risk factors among children in 4 health areas of Kisantu health zone.* Methods*. A cross-sectional study was carried out in 4 health areas of Kisantu health zone. 388 children randomly selected were screened for* S. mansoni* using Kato Katz technique and the sociodemographic data was collected. Data were entered and encoded using software EpiData version 3.1. Analysis was performed using SPSS version 21 software.* Results*. The prevalence of* S. mansoni* was 26.5% (103); almost two-thirds (63) (61.2%) had light infection intensity. A significant association was found between* S. mansoni* infection and age (*p* = 0.005), educational level (*p* = 0.001), and practices of swimming/bathing (*p* < 0.001) and using water from river/lake/stream for domestic use (*p* < 0.001). Kipasa health area had high prevalence of schistosomiasis (64.6%) (64/99; 95% CI 54.4–74.0) compared to other health areas.* Conclusion*.* Schistosoma mansoni* infection still remains a public health problem in these areas. There is a need to promote health education and promote behavioral changes in children towards schistosomiasis.

## 1. Introduction

Worldwide, schistosomiasis is still a global health problem in the 21st century. It continues to threaten millions of people, particularly in Sub-Saharan Africa [1, 2]. By its prevalence, schistosomiasis ranks 1st of diseases transmitted by water and has 2nd place for its public health importance in tropical and subtropical areas of the globe, behind malaria [3]. It is estimated that 600 million people are at risk of schistosomiasis. Among them 200 million people in 74 countries are infected, of whom 120 million are symptomatic and 20 million people have severe disease; 85% live in Sub-Saharan Africa [4, 5].

School children constitute a high risk group and are the worst affected by schistosomiasis [6]. The standard curves of prevalence of age for* Schistosoma mansoni*, which are based on the excretion of eggs, show that the prevalence and intensity of infection peak are between 10 and 15 years, after which the prevalence decreases gradually over the years and intensity of infection declines more rapidly [7]. The age distribution of the prevalence and intensity of infection are usually attributed to the high levels of contact with contaminated water cercariae in school aged children and adolescents followed by less contact with water and the development of a protective acquired immunity against infection in older adolescents and in adulthood [8, 9].

Globally, in the African Region of WHO, 10 countries account for 67.4% of the total number of people that require preventive chemotherapy [10]. Democratic Republic of the Congo (DRC) is one of the endemic countries for schistosomiasis which WHO has ranked among countries where preventive chemotherapy is required [10]. Schistosomiasis has been found present in some provinces for over a century [11]. However, there is a particular lack of surveillance activity related to schistosomiasis in the DRC. Kongo Central (Bas-Congo) is one of the endemic provinces for schistosomiasis in the DRC as highlighted by Madinga et al. [12]. At present, there are only estimates of the disease burden of schistosomiasis which are inaccurate due to lack of studies and these authors emphasize that there is an urgent need to investigate the prevalence of neglected tropical diseases [13].

## 2. Materials and Methods

The pilot survey was carried out from January 2016 to February 2016 in Kisantu health zone (KHZ) situated in the province of Kongo Central in the western part of Democratic Republic of the Congo, with a rainy season from September 15 to May 15 and a dry season from May 15 to September 15 of each year. The population of KHZ is estimated at 183,749 inhabitants, whose principal activity is agriculture. The weakness of agriculture does not allow the population to cover the needs of 100% of the population of KHZ [14]. The major causes of morbidity remain the unhealthiness, promiscuity, lack of latrines, and social debridement with increasing of sexually transmitted infections [14]. KHZ is crossed by several rivers including the Inkisi River, Ngufu River, Lassa River, Wolokoso River, Luwuwa River, and Kiela River. These rivers contain snails, intermediate hosts of schistosomes. The most snail species found along these rivers are* Biomphalaria pfeifferi*; however* Bulinus forskalii* are also reported [14, 15]. The more prevalent water is free flowing, with a low flow rate, which promotes domestic use of this water. However, there is also stagnant water.

KHZ is composed of 15 health areas, rural, semirural, and urban. In total 4 health areas (HA) were selected based on the level of the degree of urbanization. These 4 HA were Kipasa rural HA crossed by rivers Kiela and Lassa, Nkandu semirural HA, Kitanu 1 urban HA, and Kitanu 2 semirural HA.

The study population was randomly selected within the community from these 4 health areas. A total of 388 children participated in the study by responding to a questionnaire and providing the required faeces samples.

The parasitological examination was performed by the method of Kato Katz [16] in the laboratory of Tropical Medicine/Faculty of Medicine of University of Kinshasa. It had a faeces sample obtained by means of a spatula. It was passed through a sieve to remove the coarse particles. 41.7 mg of these faeces was collected using a mold and placed on a slide glass and then covered with a piece of cellophane that has kept for at least 24 hours in a glycerin solution with malachite green. The blade was turned so that the covered sample of cellophane is placed against a flat surface. It correctly makes straight smear; reading and counting were made at ×400 magnification of the optical microscope. The number of eggs found was multiplied by 24 in order to obtain the number of eggs per gram of feces (EPG). According to WHO guidelines, parasite egg counts were used to classify* Schistosoma mansoni *infection into light (1–99 EPG), moderate (100–399 EPG), or heavy infection intensity (≥400 EPG) [6].

Data were entered and encoded using software Epidata version 3.1. Analysis was performed using SPSS version 21 software. Descriptive statistics were presented as mean ± standard deviation for the continuous data and percentages for categorical data. Pearson's chi-square was used to compare proportions. *p* values less than 0.05 were considered statistically significant.

### 2.1. Ethical Considerations

Ethical approval was provided by Ethics Committee of the School of Public Health of the Faculty of Medicine/University of Kinshasa (approbation number ESP/CE/077/15) in Democratic Republic of the Congo. Before inclusion, written informed consent was obtained from the parents or legal tutor. Each informed consent was signed. For those who did not know how to sign for any reason, we took a thumb print instead of the signature.

## 3. Results

In total, three hundred and eighty-eight (388) children from 10 to 18 years with a mean age of 12.7 ± 2.05 years were included in the study. Of the 388 children examined, 212 (54.8%) were males while 176 (45.2%) were females. From these 388 children examined, 103 (26.5%) were infected with* S. mansoni* and 54 (52.4%) children were males versus 49 (47.6%) females. Of 103 children infected by* S. mansoni*, almost two-thirds (66) (64%) were in the age group of 10–12 years, 31 (30%) were in the age group of 13–15 years, and 6 (6%) were in the age group of 16–18 years. Among these infected children, 63 (61.2%) were found with a light infection intensity while 25 (24.3%) had a moderate infection and only 15 (14.5%) children had a heavy infection intensity. Schistosomiasis was associated with age (*p* = 0.005) and educational level (*p* = 0.001). According to educational system of Democratic Republic of the Congo, primary school is from grade 1 to grade 6 and secondary school is from grade 7 to grade 12. The mean time taken by children from home to river/lake/stream was 16.68 minutes with minimum time of 1 minute and maximum of 120 minutes. Schistosomiasis was significantly associated (*p* < 0.001) with practices of swimming/bathing in open water (*p* < 0.001) and of using water from river/lake/stream for domestic use, but it was not associated with profession of father (*p* = 0.09) and mother (*p* = 0.08) who were largely farmers (Table 1). Children of Kipasa health area had high prevalence of schistosomiasis (64.6%) (64/99; 95% CI 54.4–74.0) compared to other health areas (Table 2).

## 4. Discussion

This descriptive study showed that schistosomiasis still remains a real public health problem in the four health areas. Schistosomiasis prevalence rate was 26.5% (103).

The findings are close to those reported in Songololo Territory, Democratic Republic of the Congo (31.2%) [17], and in Nigeria (32.2%) [18] while that is less than those found in several studies in Tanzania (64.3%) [19], (68%) in Mali [20], and (82.7%) in Kasansa health zone, Democratic Republic of the Congo [21]. However, that is higher than those found in other studies elsewhere, 6.4% in Mokali health area, Democratic Republic of the Congo [22], and 8.0% and 9.3% in Yemen [23, 24].

The prevalence was similar in both genders (25.5% for males and 27.8% for females) with *p* = 0.59. In DRC, especially in KHZ, males are more in contact with water due to their habits of swimming/bathing in this open water; however, females help their mother in household activities and usually go to water for domestic activities such as laundry and doing dishes. This could explain the fact that both male and female are equally infected. These findings are similar to those found by several authors, especially in Mali [20], in Kenya [25], in Ethiopia [26], and in Democratic Republic of the Congo [21]. However, the results differ from several studies where male predominance was reported in Yemen [24], in Ethiopia [27], and in Ghana [28].

The intensity infection showed that the largest number of infected children (61.2%) (63) had light intensity. The findings are similar to those reported in Ethiopia [27, 29], in Democratic Republic of the Congo [22], and in Yemen [23]. However, these results are opposite to those found in Nigeria [18] and in Ethiopia [26, 30].

The findings showed that schistosomiasis was associated with age and educational level. Montresor et al. emphasized that children are more vulnerable and susceptible to infection because of their poor hygiene and playing habits in the water [31]. Most infected children were in age group 10–12 years. Younger children are those who have most habits to play/swim/urinate/defecate into the water and being close to water river/lake/stream increases this risk of contracting schistosomiasis. These results are close to those in Nigeria [32], in Senegal [33], in Ethiopia [26], and in Tanzania [19]. However, other authors found different results in Ethiopia [34] and in Côte d'Ivoire [35]. Association between schistosomiasis and educational level was also reported in Ethiopia [29].

The mean time taken by children to reach river was 16.68 minutes; it means that they were close to the water and being close to water increases the risk of contracting schistosomiasis as that was reported in Zambia [36], in Côte d'Ivoire [37], and in Yemen [24].

The findings showed that schistosomiasis was significantly associated with practices of swimming/bathing in open water (*p* < 0.001); these results are similar to those found in Ghana [28], Nigeria [18], and Ethiopia [29].

The findings also showed that schistosomiasis was significantly associated with practices using water from river/lake/stream for domestic use (*p* < 0.001); just 27.6% never used this water at home. These results are similar to those found in Nigeria [18, 32], in Kenya [38], in Yemen [24], and in Ethiopia [27, 29, 30].

Although farming activities are often associated with schistosomiasis, the findings showed that the prevalence was not associated with farming activities of parents (*p* = 0.09 for father and *p* = 0.08 for mother); these results are similar to those found in Côte d'Ivoire [37] and in Yemen [24]. However, other studies found the opposite, as in Ethiopia [27, 34] and in Ghana [28].

Children of Kipasa health area had high prevalence of schistosomiasis (64.6%) (64/99; 95% CI 54.4–74.0) compared to other health areas. Unfortunately, this study did not assess the factors associated with infection that could explain this difference. However, this high rate could be explained by the fact that this health area is crossed by two rivers, the Lassa River and the Kiela River, which are incriminated as shelter for snails, intermediate hosts of* Schistosoma* [15], and children living there are more closely in contact with these rivers and therefore more at risk of being infected by schistosomiasis compared to children living in the other three health areas.

## 5. Conclusion

The study conducted among children in Kisantu health zone revealed that schistosomiasis remains a major public health problem. Schistosomiasis was associated with age, educational level, and the practices of swimming/bathing/using water from river for domestic use and Kipasa health area had a high prevalence.

Therefore, there is a need to promote health education of children and parents, to enhance communication for behavior change towards schistosomiasis and to promote the fight for the elimination of snails.

## Figures and Tables

**Table 1:** *Schistosoma mansoni* infection and associated factors in the four health areas of Kisantu health zone.

Variables	*Schistosoma* presence				Total number	Total percent	*χ* ^2^	*p* value
	Positive		Negative					
	Number	Percent	Number	Percent				
*Gender*								
Male	54	25.5	158	74.5	212	54.8	0.27	0.59
Female	49	27.8	127	72.2	176	45.2		

*Age*								
10–12	66	33.5	131	66.5	197	50.8		
13–15	31	20.8	118	79.2	149	38.4	10.64	0.005^*∗*^
16–18	6	14.3	36	85.7	42	10.8		

*Educational level*								
Primary	90	31.7	194	68.3	284	73.2		
Secondary	12	12.2	86	87.8	98	25.3	14.43	0.001^*∗*^
Not at school	1	16.7	5	83.3	6	1.5		

*Swim/bath in open water*								
Never	35	16.7	175	83.3	210	54.1		
Rarely	26	31.0	58	69.0	84	21.7	27.2	<0.001^*∗*^
Always	42	44.7	52	55.3	94	24.2		

*Using water from river/lake/stream for domestic use*								
Never	26	24.3	81	75.7	107	27.6		
Rarely	30	18.0	137	82.0	167	43.0	19.18	<0.001^*∗*^
Always	47	41.2	67	58.8	114	29.4		

*Profession of father*								
Farmer	73	29.70	173	70.30	246	63.40	2.95	0.09
Other	30	21.10	112	78.90	142	36.60		

*Profession of mother*								
Farmer	87	28.80	215	71.20	302	77.80	3.07	0.08
Other	16	18.60	70	81.40	86	22.20		

*Total*	*103*	*26.5*	*285*	*73.5*	*388*	*100.0*		

^*∗*^Significant association *p* < 0.05.

**Table 2:** Prevalence of *Schistosoma mansoni* infection in the four health areas of Kisantu health zone.

Heath area	Number		Percent	95% CI
	Examined	Infected		
KIPASA	99	64	64.6	54.4–74.0
NKANDU	98	11	11.2	5.6–18.8
KITANU 1	95	21	22.1	14.2–31.9
KITANU 2	96	7	7.3	3.1–14.7

*Total*	*388*	*103*	*26.5*	*22.3–31.3*
